# Supplementary material for: Applying heat and humidity using stove boiled water for decontamination of N95 respirators in low resource settings
Source: PLoS One. 2021 Sep 30;16(9):e0255338. doi: 10.1371/journal.pone.0255338 (PMC8483377; doi:10.1371/journal.pone.0255338)
Supplement: S1 File — (PDF) [file pone.0255338.s001.pdf]

S1 File

Applying Heat and Humidity using Stove Boiled Water for Decontamination of N95 Respirators in Low Resource Settings

Siddharth Doshi[1], Samhita P. Banavar[2], Eliott Flaum[2,3], Surendra Kulkarni[4], Ulhas Vaidya[4], Shailabh Kumar[2], Tyler Chen[2], Arnab Bhattacharya[4], Manu Prakash[2]*

[1]Department of Materials Science and Engineering, Stanford University, Stanford, CA, USA
[2]Department of Bioengineering, Stanford University, Stanford, CA, USA
[3]Graduate Program in Biophysics, Stanford University, Stanford, CA, USA
[4]Tata Institute of Fundamental Research, Mumbai, 400005, India

S1.1 Details of Heating Protocol for Experiments Conducted In Mumbai, India

Temperature and humidity readings were taken with an Elitech GSP-6 meter, with the probes taped onto the mask surface. Experiments on N95 masks were carried out in Mumbai between 14-21 July 2020, with the ambient temperature and humidity varying between 28-32°C and 75-92%, respectively. Tissue paper was dosed with 1 ml water for the initial trials, since this resulted in humidity levels above 90%, the water content was reduced to 0.5 ml. Given the varying wind and humidity over the period of the experiment, after the first trial, a cloth was wrapped around the outer vessel to maintain the heat for a longer time and obtain better reproducibility.

Since the temperature and humidity probe wires had to come out of the vessel, sealing the inner vessel was important. Without this, the near saturated humid air in the outer vessel would get inside (as found in the first trial). This was done using aluminium foil. However, the high humidity levels had no detrimental influence on the filtration efficiency of the mask during this first trial, also illustrating the robustness of this process to changes in conditions.

S1.2 Experimental Details of Filtration Efficiency Measurements Conducted In India

A simple home-built, low-cost, compact, particle filtration efficiency setup was used to evaluate the particulate filtration efficiency at 0.3μm at a flow rate of 10 lpm. The data are shown in Fig. 4 of the main manuscript. The setup uses a Plantower PMS 7003 particle concentration sensor air quality monitor chip (which can separately measure particle counts in different size channels between 0.3-10 μm)and a ESP8266-based WiFi microcontroller. The data transmitted from the ESP8266 module to a web server is visualized and analyzed through a simple intuitive HTML-based web interface, which also has an in-built efficiency calculator. All the construction details, diagrams, source codes for the micro-controller and interface are available open-source at the GitHub repository https://github.com/shescitech/TIFR_Mask_Efficiency. A schematic diagram of the setup is shown in Fig. S1.

A background of fine particles was generated using normal saline solution in a standard medical nebulizer to create a fine aerosol. Air was sucked through the particle counter using an oil-free diaphragm pump and the throughput of particles in the 0.3 μm channel measured with and without a mask at the input. The entire mask was placed on a plastic ball, and taped all around at the sides to exclude ingress of particles from the sides. We estimate a ±1% accuracy for measurements made with this setup.

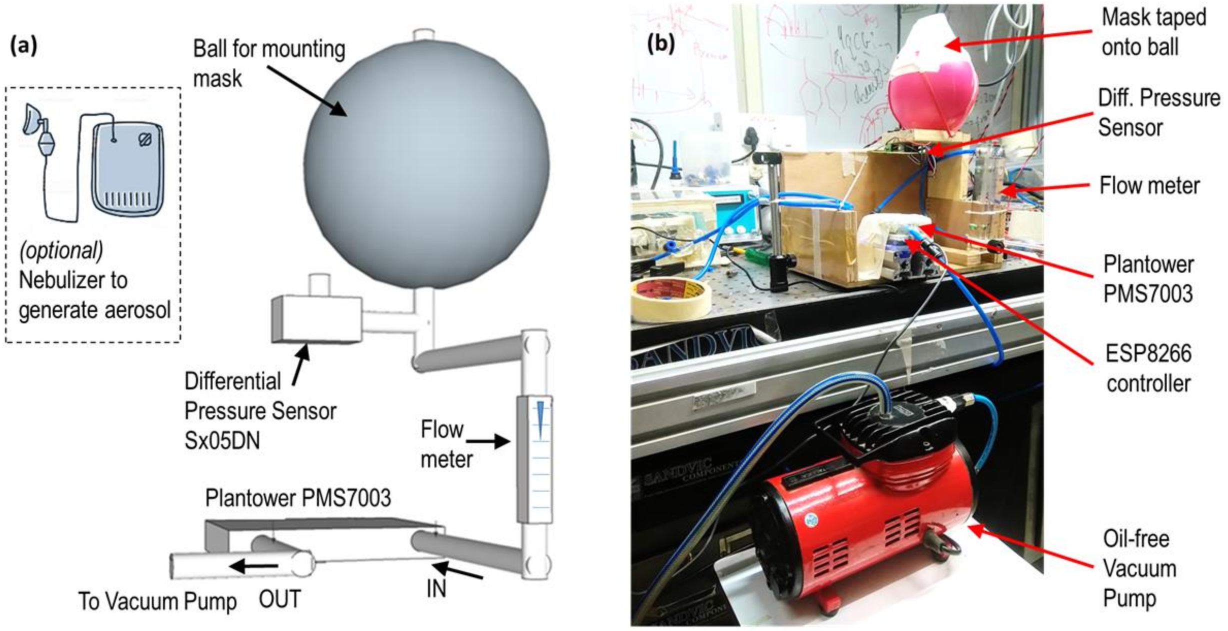

Fig. S1: Schematic diagram and picture of the experimental setup used to measure the particle filtration efficiency at TIFR Mumbai. Further details of the setup can be found at https://tinyurl.com/TIFRmasktester

S1.3 Potential Route to Scale-Up Involving Dumpling Steamers

The scalability of this setup to a multi-mask arrangement using a home “idli-steamer” was shown in Fig. 9 of the main manuscript. We suggest that this technique can easily be scaled up to much larger numbers using steamers commonly used in typical institutional canteens. Fig. S2 shows a typical large sized “idli-steamer” used in a typical Indian canteen. With small modifications, such a system could easily be configured to hold 40-50 masks (in 2 layers) for a moist-heat based decontamination process. We also note that multilayer similar steamers for making dumplings like “momos” are also common in parts of India and Asia.

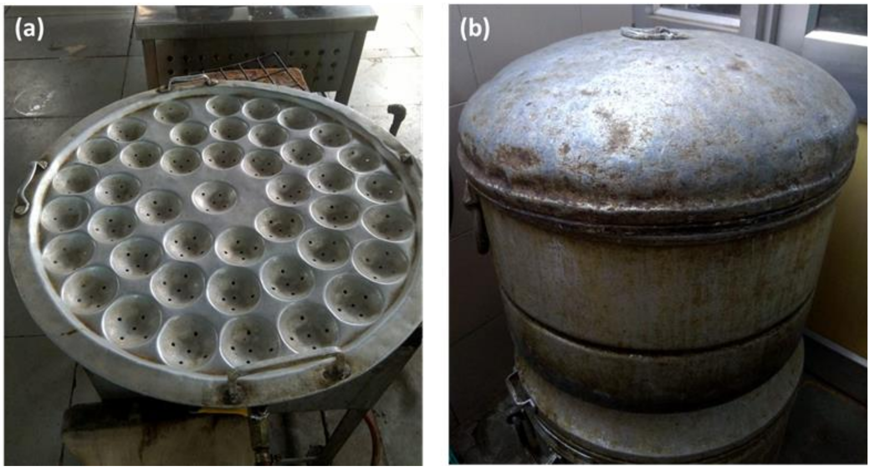

Fig. S2: (a) shows a large-scale insert and (b) shows the steamer used in a typical Indian canteen, such an arrangement can easily hold 40-50 masks in two layers.

S1.4 Experimental Details of Decontamination Experiments on Surgical Masks Conducted In Mumbai, India

In a separate set of experiments, a similar humid-heat based decontamination treatment was explored for high-quality 3-ply surgical masks containing a melt-blown polymer layer. We used a Venus 1010 surgical mask, a widely used brand in India, which is compliant to the IS:16289 Indian Standards for surgical face masks, and had a PFE of ~98-99% in the pristine state. These experiments were carried out in Oct/Nov. 2020. The same 5L aluminum outer vessel with a 2L stainless steel inner container was used for this study.

The temperature and humidity profiles for 5 different cycles are shown in Fig. S3. The measured temperature and humidity exceeded the target values in all the cycles. In most cases the temperature remained > 65 ^{o}C for ~60 minutes. Even in the one case with a potential leak in the insulation, the minimum duration of 30 minutes was achieved, showing, as in the case of the N95 masks, that the process is quite robust to small variations in the initial conditions.

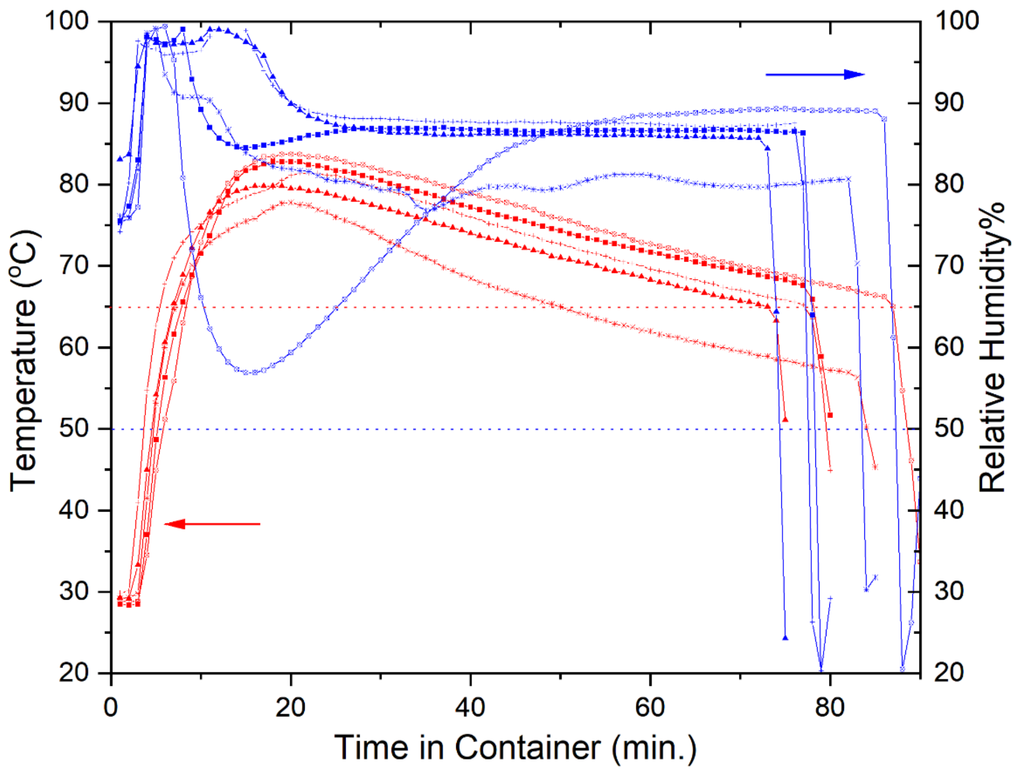

Fig. S3: Temperature and relative humidity inside the container holding the Venus 1010 surgical mask during 5 treatment cycles. The dashed lines indicate the minimum target temperature (65°C) and humidity (50%). Though there seems to be an imperfect sealing in one of the runs, the temperature still remains > 65°C for >30 minutes.

The particulate filtration efficiency at 0.3um was measured at a flow rate of 10 litres per minute for the pristine and heat-treated masks after every decontamination cycle. Within limits of experimental error, there is no degradation in efficiency due to the humid-heat treatment.

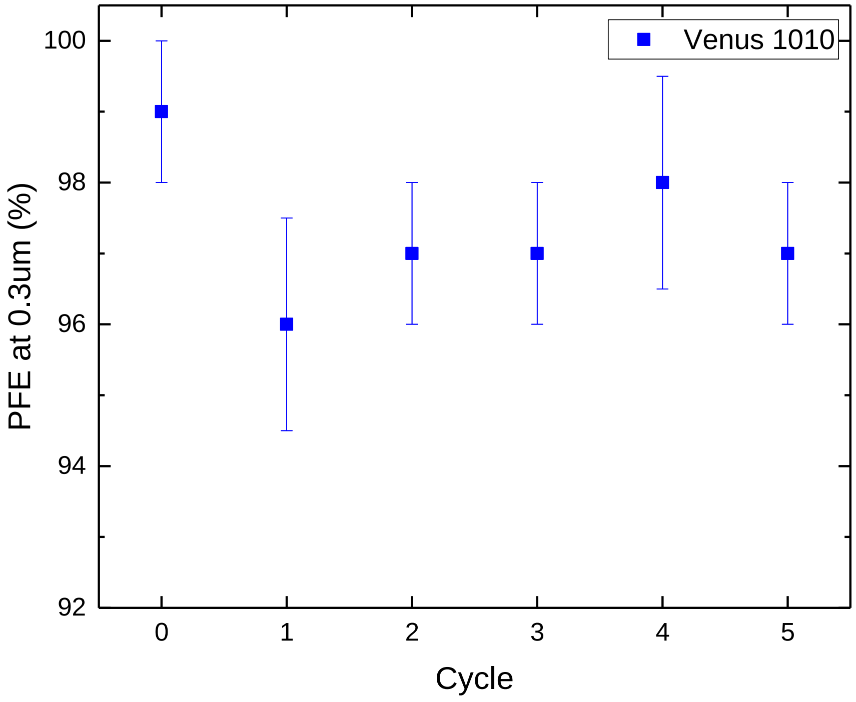

Fig. S4: Particulate filtration efficiency at 0.3 μm for the pristine surgical mask and after each decontamination and heating cycle.
